# Welcoming patients into our eye service

**Published:** 2012

**Authors:** Jonathan Pons

**Affiliations:** Ophthalmologist and Programme Director, Good Shepherd Hospital Eye Care Project, PO Box 218, Siteki, Swaziland. Email: jono@goodshepherdhosp.org

**Figure:**
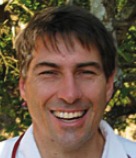
Jonathan Pons

## Going to the eye clinic?

Before our patients even leave home for the clinic or hospital, they are already anxious. They may have many questions, such as: “How much will the transport cost? Will I find someone reliable to look after my children? How early will I have to leave to get to there on time? What will they say? What will they do?”

All too often, patients’ fears are realised when they arrive at the hospital: staff are impatient because they are in the wrong queue, and they might even be shouted at or humiliated. Add to this being tired and hungry, sitting on an uncomfortable chair, and being unable to leave it for fear of losing a place in the line. Not to mention worrying about the cost, the long return trip back home, and the children. “Oh, for a kind face and someone who really cares!”

Well, we do really care, but so often other worthwhile goals such as efficiency, reducing costs, and ensuring good clinical outcomes take greater priority. In the complexity of managing a busy waiting area, improving the patient experience may well be neglected!

In this article we hope to show you how to make a patient's entry into the eye clinic as comfortable and positive an experience as possible.

## Reception

The first person our patients talk to – usually the person at the reception or registration desk – sets the tone for their visit.

**Figure:**
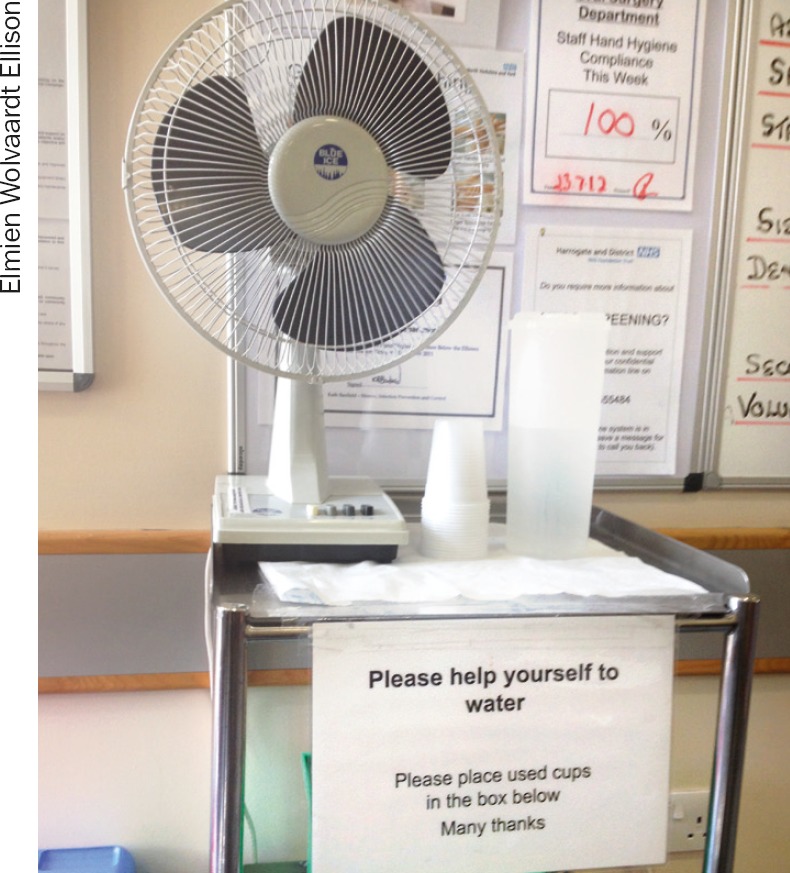
Making drinking water available shows kindness and consideration for patients.

**Figure:**
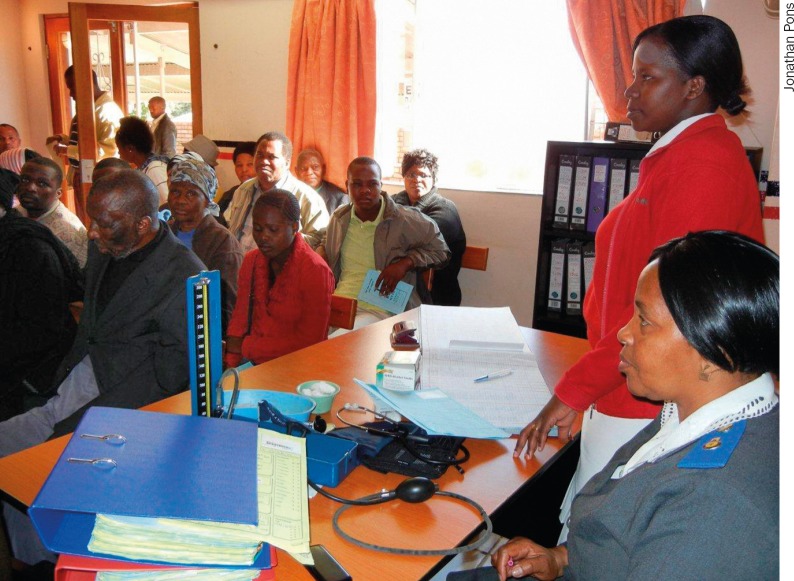
At Good Shepherd Eye Hospital, the clinic process is explained to everyone in the waiting room at the same time. SWAZILAND

It is advisable to choose someone for this role who is patient, friendly, and kind; they must also have good commucation skills and a thorough knowledge of the hospital system. They should be able to deal calmly with patients and family members who are angry or upset.

There is much to do at this stage, such as registration, retrieval of records, and so on. In addition, patients will appreciate it if the person in reception also:

- Shows them where to sit
- Points out the toilets, exits, and drinking water
- Tells them where they can get something to eat if they have to stay past lunch time
- Explains how the waiting system works – is there a queue, or should they get a ticket number? What can they do if they have to leave the room for a few minutes?

Most of all, the role of the first contact person is to reassure the patient that they have come to a professional, efficient, and caring organisation.

## Triage

Walk-in patients, especially those with pain or a visible eye injury, should be quickly assessed by a highly experienced nurse so they can be moved to the front of the queue or directed to the correct waiting area. This process is known as ‘triage’. Some eye clinics have a separate reception area for emergency patients.

Explain to other waiting patients that emergency patients always receive priority, otherwise they may worry that they have been forgotten.

## The physical environment

The waiting areas, and the corridors leading up to them, should be well lit and clearly signposted.

Paint high-visibility lines on walls and stairs; this will help patients with low vision to find their way around the building.

Pathways should be designed so they are appropriate for people in wheelchairs or people who are blind. Provide rubbish bins and tidy these areas at least once a day to ensure people will not stumble over furniture, toys, or rubbish.

It is a good idea to wipe down surfaces in the waiting area with a mild soap solution to reduce the possibility of cross-infection between patients.

Regularly maintain the chairs and seating in the waiting room. Are they comfortable and safe? Provide back support if possible, or place benches against a wall.

## Educational opportunities

An eye clinic is a good place to provide eye care information or education. Posters and brochures explaining common conditions, how they can be prevented, and how they are managed at your clinic can be very helpful. An educational or promotional audiotape or DVD with the same information is even more appropriate in a clinic serving people with visual impairment.

## Sanitation

Inspect the toilets and hand washing areas regularly. Dirty sanitation facilities are not only unhealthy, but can affect the reputation of an eye clinic! Ensure there is always toilet paper or water, depending on local preference. Provide hand soap and paper towels for drying hands.

The toilets are also a good place to add information posters about eye care and to remind patients and the people accompanying them about the importance of hand washing.

## Managing queues

There is no single answer to how best to organise queues in your clinic. Each culture has its own waiting temperament and social conventions. In many cultures, the approach to waiting is ‘first come, first served. This is usually more important than social status or even the severity of the present condition (except for emergencies). In some cultures, the very young or very old are afforded special privileges and allowed to go to the front of the queue.

However, whatever the local conventions, one of the worst things about waiting is when you have no idea how long you will have to wait. This can make you feel trapped! Do what you can to keep people informed of the approximate waiting time and reassure them that the system is fair and that everyone will be seen. For example, you could give patients a numbered card when they arrive, with an approximate time when they will be seen. This allows them the freedom to use the toilet or find refreshments without losing their place in the queue.

It may save time and effort if patients are taken to the correct waiting areas by a clinic helper. This will be especially important for specialist clinics and review or post-operative services.

Reassure patients who are waiting and give them updates on how long it will take for them to be seen. A waiting room can be a tense and lonely place for an anxious patient, and if nothing seems to be going on, patients may feel they have been forgotten.

Explain what patients can expect inside the examination room. The examination room is where the patient's expectations and levels of anxiety will be most intense.

The clinic is be an intimidating environment for patients, especially children and those who are blind. Clinicians, and the health care workers who assist them, can help by giving patients and their relatives as much verbal and physical reassurance as possible.

## Using volunteers

Our eye services are generally short of human resources.

Health care workers are usually busy at their work stations and have little opportunity to help in the waiting room. So the minor needs of waiting patients are easily neglected. If there is no accompanying relative, who will help the elderly to their feet, lead patients who are blind, or clean up a mess?

**‘Inspect toilets and hand washing stations regularly. Clean facilities are safer and improve your reputation’**

Some hospitals encourage and make use of volunteers. Volunteers could come from a service organisation, a church, ora local charity. Their efforts can include running a tuck shop, fundraising, or organising support groups and eye care awareness days.

Many volunteers or their relatives have been affected by visual impairment and have benefited from the hospital services – they are sometimes motivated to improve the conditions in the hospital as a result of their own experiences.

## Make the most of opportunities

Private practice or VIP waiting rooms serve those who would like to pay for fast-track and premium services.

This has the advantage of reducing the number of people in the general waiting room and earning an income to support patients who are unable to pay. The ‘fee-for-service’ concept has helped many eye units gain financial independence.

However, it is highly advised that a VIP waiting room be housed in a different building, to avoid offending those who may have to wait long hours. If this is not possible, consider constructing a separate entrance into the main eye clinic for the “fast-track patients.

**TIPS:**

- “In our clinic, we have TV entertainment, a drinks machine, and also a moving-print message board saying what the delay time is, etc.”
- “We have a whiteboard which has the names of the clinic staff, together with their title and role.”
- “To prevent misunderstandings, we have a notice in casualty saying that urgent patients may need to be seen out of sequence”
- “We allow a community lady to sell *vetkoek* (fried sweet bread) to our patients.”
- “We have two types of patients coming every day: new patients and patients coming for review. We give patients numbered cards to put them in order as they arrive, and we use different colours for the different types. Patients coming for review get red number cards so that they can easily identified and seen faster. This decongests the system since they will not need to go through all the services. Of course emergencies are identified at the reception and handled accordingly.”

*With many thanks to Faustin Dennis Ngounou and staff members at Good Shepherd Eye Hospital for their contributions of practical tips and ideas*.

**The case for a walk-in clinic**

At Good Shepherd Eye Hospital, we have abandoned appointment clinics in favour of walk-in clinics. This benefits both ourselves and our patients.

**Table:** 

Patient reasons:	Eye clinic reasons:
**1** ‘First-come, first-served’ is considered fair	**1** Human resources saved as no clerk needed to take appointments
**2** No need to make arrangements	**2** Complex lists and organisation avoided
**3** May combine with visit to other hospital clinics	**3** Patient delays or cancellations do not affect the eye service
**4** Assured of access in case of emergency	**4** The competitive nature amongst patients ensures an early start to the clinic.	
**5** Own decision to attend clinic may change after observing a full or empty clinic.	

